# Design of Synchronization Tracking Adaptive Control for Bilateral Teleoperation System with Time-Varying Delays

**DOI:** 10.3390/s22207798

**Published:** 2022-10-14

**Authors:** Kesong Chen, Haochen Zhang

**Affiliations:** 1 College of Electrical and Information Engineering, Lanzhou University of Technology, Lanzhou 730050, China; 2 Gansu Provincial Key Laboratory of Advanced Industrial Process Control, Lanzhou 730050, China

**Keywords:** teleoperation system, adaptive control, position tracking, velocity feedback filter, time-varying delay

## Abstract

The performances of position synchronization and force interaction of the teleoperation system provide a safe and efficient way for operators to perform tasks in remote, hazardous environments. In practice, however, communication delays and dynamic uncertainties can impair the performance of position synchronization controls. Under the above factors, it is necessary to study and design appropriate bilateral control methods to achieve stable and effective position synchronization control. In this paper, a new adaptive control architecture based on velocity feedback filter and radial basis function neural network is proposed. In the proposed control scheme, only the position signal is transmitted during the communication process, and the speed feedback filter and compensation method are designed and adopted to avoid the use of acceleration signals. In addition, a new auxiliary variable with a tracking error integral term is used to reduce the steady-state error of position tracking under nonzero external environmental forces. Using the Lyapunov–Krasovskii method, the stability of closed-loop remote operating systems is demonstrated. In the simulation and experiment sections, the algorithm was verified separately and compared with other algorithms. The results of a master–slave robot system verify the tracking performance of our proposed control scheme.

## 1. Introduction

The biological laboratory has very high requirements for cleanliness, and operators must implement three-level protection before entering for operation. Using teleoperation robots to replace humans to work in the high cleanliness laboratory can avoid the operator from polluting the experimental area and affecting the experimental results. The teleoperation system is a typical network robot system, which has also been widely practiced in many engineering fields, such as space exploration [1], deep-sea operation [2], telesurgery [3], disposal of hazardous materials [4], and so on. In the teleoperation system, the human operator can control and operate the remote slave robot through the master robot and force feedback devices. The force of the slave robot generated by contacting with the task environment can also be feedback to the human operator. Between the master and slave robots, the position, speed, and other information of the robots are transmitted through the communication channel to realize the information interaction and the position synchronization control. It is well known that communication will lead to signal transmission delay, which will weaken the performance of position synchronization control of the teleoperation system and even cause system instability. Therefore, achieving stable and high-performance master–slave position tracking is one of the important indicators for the teleoperation system.

Many research works have been studied to improve tracking performance in practice [5]. For one thing, as the signals of the local robot and remote robot are transmitted by the communication channel, the communication delays in the transmission cannot be evitable. Communication delay, especially asynchronous communication delay, can reduce the control performance of the teleoperation system and even cause system instability [6]. For another, it is well known that the robot model is nonlinear and time-varying dynamic. Especially, the friction, high-frequency model parameter perturbations, uncertain external disturbances, and unknown environment descriptions are difficult to estimate and identify, which can impede the improvement of operating performance in the teleoperation.

For decades, some classic methods are proposed to deal with the adverse effects of time delays, such as scattering approach [7,8], wave variable method [9,10], PD control [11,12], sliding mode control [13,14,15], $H_{\infty }$ control [15], output feedback control [16,17], feedforward-feedback position control [18], and observer approach [4,19]. These control algorithms are based on passive theory, Lyapunov theory, and the main force on the stability of the system with time delays. The uncertainties of teleoperation dynamics have not been adequately considered.

Adaptive control is a more effective method for improving the performance of a complex nonlinear system with poor structure dynamics and unknown disturbance. Some researchers have applied the adaptive method to the teleoperation system control. The main principle of these adaptive methods is to use the fuzzy logic system, Radial Basis Function (RBF) neural network, and linear parametric model to estimate the uncertain model and external interference.

In [20], a novel fuzzy adaptive control method is proposed, the dynamic uncertain parts are defined as a nonlinear function, and the fuzzy system is applied to estimate it. In [21,22], the adaptive linearly parameterizable method is employed to estimate the uncertainties parts and time-varying delay is considered. In [22], two adaptive control schemes are developed for position tracking of master–slave robots. The RBF neural network is applied to approximate the model uncertainties. In [23], the robust mechanism is introduced to enhance the robustness of the system and the adaptive controllers are developed for master and slave robots. In [24], the uncertainties in both kinematics and dynamics of the teleoperation system are considered, and an adaptive control approach based on the fuzzy logic system is developed.

The above research works are mainly focused on the position tracking control of the teleoperation with fixed time delays, and time-varying delays have not been fully considered. Furthermore, researchers have paid more attention to the position tracking of the teleoperation with time-varying delays, which has more practical engineering significance. At the same time, the parametric uncertainties are also taken into account. In [25,26], Yang and Li investigate two adaptive control schemes based on multiple fuzzy logic systems for teleoperation systems. In [27], Yang et al. introduce the RBF neural network and propose a new adaptive wave variable structure for the teleoperation system with time delays. In [28,29], two bilateral control schemes based on type-2 Takagi Sugeno (T-S) fuzzy system are proposed for position tracking of the teleoperation system. The T-S fuzzy model is utilized to represent the dynamic for controller design and guarantee the motion synchronization when time-varying delays and uncertainties exist. In [29], the authors address the problem of task motion tracking in the teleoperation system with uncertain dynamic/kinematic and time-varying delays. A new adaptive controller and related adaptive laws are designed. Huang et al. [30] develop a new adaptive sliding mode controller based on the projection mapping method, and the adaptive laws and control laws are designed for parameter estimation and position tracking control. In [31,32], the signal smoothing filter is introduced into the communication process from the master robot to the slave robot to smooth the position information transmitted with time delay and enhance the stability of the system. In [33,34], adaptive fixed-time control methods for position tracking of the teleoperation system are presented to implement fast position error convergence performance.

It is noted that the auxiliary variable and sliding surface in the above works are composed of tracking error and error differential. The steady-state error of motion tracking may exist under the active operation forces and external interference acting. In [35,36], the integral term of position tracking error is introduced to provide more accurate motion tracking. In [35], an integral sliding mode control structure is proposed for synchronization control. In [36], a novel finite-time control based on the error integral is developed. However, there are still problems in the above research works: for one thing, the position acceleration signals are used to calculate the control laws and estimate the uncertain parts of the teleoperation system; for another, in the above control structure, both position and velocity signals are transmitted through the communication network, which increases the amount of data transmitted by the signal. If only the position information is transmitted and the velocity information is obtained by deriving the position signals at the controller side, the amount of data transmitted can be reduced. However, the change effect of delay is also superimposed in the position and speed information, which may weaken the control performance of the system.

Therefore, following the line of challenges in the above works, this paper mainly solves the problem of position tracking control of the teleoperation system in the presence of time-varying delays and uncertain models. At the same time, the proposed new control scheme can improve position tracking accuracy and avoid the use of acceleration signals. The main contributions of our work can be summarized as follows:

- A novel auxiliary variable constructed by position error integral term is proposed, and a new adaptive control scheme for teleoperation system is also developed. Compared with the existing auxiliary variables constructed by errors and error differential terms, the proposed auxiliary variables and control method have better robustness and smaller steady-state error.
- The new velocity feedback filter and velocity delay adaptive compensation are designed to avoid the use of acceleration signals and improve the stability of the system. On the other hand, only the position information is transmitted by communication to reduce the amount of communication data, and the velocity is obtained by calculating the time derivative of the position at the controller side. In this way, the time delay information will be added to the velocity signals. If the communication time delay changes greatly, it will produce a large time delay superimposed on the velocity information and weaken the stability of the system. Therefore, we design and introduce an adaptive compensation term of velocity delay to improve the stability of the system.

The remainder of this paper is presented as follows. In Section 2, the dynamic descriptions of the teleoperation system are described and some preliminary definitions are given. In Section 3, the proposed adaptive control scheme is investigated. Subsequently, in Section 4, the stability analysis is discussed. The simulation and experiment results and illustrations are given in Section 5. Finally, this work is concluded in Section 6.

## 2. Problem Formulation

### 2.1. Master–Slave Telerobotic System

In this paper, the dynamic description of a telerobotic system based on the Euler–Lagrange method are given as

(1) $$\left\{\begin{array}{cc} \; & M_{m}\left(q_{m}\right){\ddot{q}}_{m}+C_{m}\left(q_{m},{\dot{q}}_{m}\right){\dot{q}}_{m}+G_{m}\left(q_{m}\right)+B_{m}=\tau_{m}+\tau_{h},\\ \; & M_{s}\left(q_{s}\right){\ddot{q}}_{s}+C_{s}\left(q_{s},{\dot{q}}_{s}\right){\dot{q}}_{s}+G_{s}\left(q_{s}\right)+B_{s}=\tau_{s}-\tau_{e}. \end{array}\right.$$

where both the master and slave robots are revolute joint manipulators with n Degree of Freedom (DOF). The subscripts {m, s} represent the master robot and slave robot, respectively. For j=m,s, $M_{j}\left(q_{j}\right)\in R^{n\times n}$ is the mass inertia matrix; $C_{j}\left(q_{j},{\dot{q}}_{j}\right)\in R^{n\times n}$ is the coriolis and centripetal matrix; $G_{j}\left(q_{j}\right)\in R^{n}$ is the vector of gravitational torque; $B_{j}\in R^{n}$ is the vector of friction torque; $q_{j}\in R^{n}$, ${\dot{q}}_{j}\in R^{n}$, and ${\ddot{q}}_{j}\in R^{n}$ are the vectors of joint position, joint velocity, and joint acceleration, respectively; $\tau_{j}\in R^{n}$ is the input control torque vector; $\tau_{h}\in R^{n}$ and $\tau_{e}\in R^{n}$ are the external torque vectors acted by the human operator and task environment, respectively. In our work, the external environment torque $\tau_{e}$ is defined as the spring damper model, which is described as $\tau_{e}=D_{e}{\dot{q}}_{s}+S_{e}q_{e}$. $D_{e},S_{e}\in R^{n\times n}$ are the unknown damping matrix and spring matrix of the environment.

There are some properties and assumptions of teleoperation dynamics in (1), which are as follows:

**Property** **1.** *The inertia matrix $M_{j}\left(q_{m}\right)$ is the positive and symmetric definite matrix. Moreover, $\exists \lambda_{j,min}$ and $\exists \lambda_{j,max}$, there is $0<\lambda_{j,min}I_{n\times n}\leq M_{j}\left(q_{m}\right)\leq \lambda_{j,max}I_{n\times n}$.*

**Property** **2.** *${\dot{M}}_{j}\left(q_{m}\right)-2C_{j}\left(q_{s},{\dot{q}}_{s}\right)$ is the skew symmetric matrix, and for any vector $y\in R^{n}$, the following equation always holds:*

$$y^{T}\left({\dot{M}}_{j}\left(q_{m}\right)-2C_{j}\left(q_{s},{\dot{q}}_{s}\right)\right)y=0.$$

**Assumption** **1.** *In a teleoperation system, the signals of master and slave robots are transmitted to each other by the communication channels. We define $d_{m}\left(t\right)$ as the communication delay from the master robot to the slave robot, and $d_{s}\left(t\right)$ as the time delay from the slave robot to the master robot. In addition, as $d_{m}\left(t\right)$ and $d_{s}\left(t\right)$ are both time-varying, bounded, and asynchronous delays, we can have $0\leq d_{m}\left(t\right)\leq {\bar{d}}_{m}$, $0\leq d_{s}\left(t\right)\leq {\bar{d}}_{s}$, $|{\dot{d}}_{m}\left(t\right)|\leq D_{m}$, and $|{\dot{d}}_{s}\left(t\right)|\leq D_{s}$.*

**Assumption** **2.** *The human operator torque $\tau_{h}$ and environment torque $\tau_{e}$ are all bounded, and we have $∥\tau_{h}{∥}^{2}\leq {\bar{\tau }}_{h}$ and $∥\tau_{e}{∥}^{2}\leq {\bar{\tau }}_{e}$.*

In the practical system, there may be uncertain dynamic parts caused by inaccurate parameters in the teleoperation system, such as $M_{j}\left(q_{m}\right)$, $C_{j}\left(q_{s},{\dot{q}}_{s}\right)$, and $G_{j}\left(q_{j}\right)$. To deal with the uncertainties, we introduce the nominal models and uncertain models to describe the $M_{j}\left(q_{m}\right)$, $C_{j}\left(q_{s},{\dot{q}}_{s}\right)$, and $G_{j}\left(q_{j}\right)$ as

(2) $$\left\{\begin{array}{cc} \; & M_{j}\left(q_{j}\right)=M_{jo}+\Delta M_{j},\\ \; & C_{j}\left(q_{s},{\dot{q}}_{s}\right)=C_{jo}+\Delta C_{j},\\ \; & G_{j}\left(q_{j}\right)=G_{jo}+\Delta G_{j}. \end{array}\right.$$

### 2.2. The Definition of RBF Neural Network

The RBF neural network has been widely applied in the controller design of the uncertain model system, with its property that the RBF neural network can approximate any nonlinear smooth function with arbitrary precision [37,38]. RBF neural network has three layers, namely, the input layer, the hidden layer, and the output layer. Based on its excellent characteristics, RBF neural network is used in control theory, data prediction such as electric load forecasting, fault diagnosis, classification, and so on.

For a continuous function $F\left(x\right):R^{a}\to R^{f}$, based on RBF neural network it can be rewritten as

(3) $$F\left(x\right)=\theta^{T}\varphi (x)+\varepsilon .$$

where $\theta \in R^{d\times a}$ is the weight matrix and *d* is the number of network nodes. $x\in R^{a}$ is the input of RBF neural network. φ(x) is the Gaussian basis function vector and can be calculated as

(4) $$\varphi (x)=e^{-\frac{{\left(x-b_{i}\right)}^{T}\left(x-b_{i}\right)}{2c^{2}}},i=1,2,\ldots ,d.$$

$b_{i}\in R^{a}$ is the Gaussian center vector of the *i*-th node. *c* is the width of Gaussian function. ε is the bounded approximate error.

In more RBF neural network applications, there are some methods to obtain the center vector and width of the Gaussian basis function, and the weight coefficient matrix can be solved through the data training process. However, it should be noted that in the adaptive control method with RBF neural network, the weight matrix of the neural network is usually designed and obtained based on the Lyapunov stability criterion of the system. The stability and convergence of the system can be guaranteed by designing adaptive learning laws. The Gaussian center vector of the *i*-th node $b_{i}$ should be determined according to the range of the actual input value of the RBF neural network. In addition, the input data value of the RBF neural network should be within the effective range of the Gaussian function by giving an appropriate width *c*. In the following section of simulation and experiment, we also selected the center position $b_{i}$ and width *c* of the Gaussian function through the neural network input data values.

### 2.3. Control Objectives

We first define the position tracking errors $e_{m}$ and $e_{s}$ at the local side and remote side as

(5) $$\begin{array}{cc} \; & e_{m}=q_{m}\left(t\right)-q_{s}\left(t-d_{s}\right),\\ \; & e_{s}=q_{s}\left(t\right)-q_{m}\left(t-d_{m}\right). \end{array}$$

For a teleoperation system with uncertain dynamics and asymmetric time-varying delays studied in this work, the main control objective is to design the appropriate control torques $\tau_{m}$ and $\tau_{s}$ so that the closed-loop system satisfies the following performance:

- **Stability.** The teleoperation system is stable with proposed control laws, asymmetric time-varying delays, dynamic uncertainties, and bounded operator and environment torques.
- **Position tracking.** The joint position of the slave robot can track the master robot when the master robot is moving. At the same time, the master robot can also track the joint position of the slave robot, which can provide certain force feedback to the operator.

## 3. Adaptive Control Scheme Design

This section proposes a novel adaptive control scheme for the teleoperation system. First, the velocity feedback filters are designed and introduced into the master/slave control channels. We define the velocity feedback filters $\nu_{m}$ and $\nu_{s}$ as

(6) $$\begin{array}{cc} \; & {\dot{\nu }}_{m}=-\eta_{m1}\nu_{m}+\eta_{m2}{\dot{q}}_{s}\left(t-d_{s}\right),\\ \; & {\dot{\nu }}_{s}=-\eta_{s1}\nu_{s}+\eta_{s2}{\dot{q}}_{m}\left(t-d_{m}\right). \end{array}$$

where $\eta_{m1},\eta_{s1},\eta_{m2},\eta_{s2}\in R^{n\times n}$ are the positive diagonal matrix of filter.

We design a novel form of auxiliary velocity error functions $e_{m}^{\nu }$ and $e_{s}^{\nu }$ based on the introduced velocity signal feedback filter, which can be described as

(7) $$\begin{array}{cc} \; & e_{m}^{\nu }={\dot{q}}_{m}\left(t\right)-\nu_{m},\\ \; & e_{s}^{\nu }={\dot{q}}_{s}\left(t\right)-\nu_{s}. \end{array}$$

The differential operation on $e_{m}^{\nu }$ and $e_{s}^{\nu }$ can be obtained as

(8) $$\begin{array}{cc} {\dot{e}}_{m}^{\nu } & ={\ddot{q}}_{m}\left(t\right)-{\dot{\nu }}_{m}\\ \; & ={\ddot{q}}_{m}\left(t\right)+\eta_{m1}\nu_{m}-\eta_{m2}{\dot{q}}_{s}\left(t-d_{s}\right),\\ {\dot{e}}_{s}^{\nu } & ={\ddot{q}}_{s}\left(t\right)-{\dot{\nu }}_{s}\\ \; & ={\ddot{q}}_{s}\left(t\right)+\eta_{s1}\nu_{s}-\eta_{s2}{\dot{q}}_{m}\left(t-d_{m}\right). \end{array}$$

Then, we propose the novel auxiliary variables $s_{m}$ and $s_{s}$ based on the functions of auxiliary velocity error and the terms of tracking error as

(9) $$\begin{array}{cc} \; & s_{m}=e_{m}^{\nu }+\lambda_{m1}e_{m}+\lambda_{m2}\int_{0}^{t}e_{m}\;d\tau ,\\ \; & s_{s}=E_{s}^{\nu }+\lambda_{s1}e_{s}+\lambda_{s2}\int_{0}^{t}e_{s}\;d\tau . \end{array}$$

where $\lambda_{m1}$, $\lambda_{m1}$, $\lambda_{s1}$, and $\lambda_{s1}$ are the positive controller parameter matrices. Then, the derivative of the auxiliary variables $s_{m}$ and $s_{s}$ can be expressed as

(10) $$\begin{array}{cc} \; & {\dot{s}}_{m}={\dot{e}}_{m}^{\nu }+\lambda_{m1}{\dot{e}}_{m}+\lambda_{m2}e_{m},\\ \; & {\dot{s}}_{s}={\dot{e}}_{s}^{\nu }+\lambda_{s1}{\dot{e}}_{s}+\lambda_{s2}e_{s}. \end{array}$$

Based on the differential definition of $e_{m}^{\nu }$ and $e_{s}^{\nu }$ in (8), (10) can be rewritten as

(11) $$\begin{array}{cc} \; & {\dot{s}}_{m}={\ddot{q}}_{m}\left(t\right)+\eta_{m1}\nu_{m}-\eta_{m2}{\dot{q}}_{s}\left(t-d_{s}\right)+\lambda_{m1}{\dot{e}}_{m}+\lambda_{m2}e_{m},\\ \; & {\dot{s}}_{s}={\ddot{q}}_{s}\left(t\right)+\eta_{s1}\nu_{s}-\eta_{s2}{\dot{q}}_{m}\left(t-d_{m}\right)+\lambda_{s1}{\dot{e}}_{s}+\lambda_{s2}e_{s}. \end{array}$$

To simplify the descriptions of the analysis, the expression of $M_{j}{\dot{s}}_{j}$ is directly given as follows:

(12) $$\begin{array}{cc} M_{j}{\dot{s}}_{j}= & M_{j}{\ddot{q}}_{j}\left(t\right)+M_{j}\left[\eta_{j1}\nu_{j}-\eta_{j2}{\dot{q}}_{j^{\prime }}\left(t-d_{j^{\prime }}\right)+\lambda_{j1}{\dot{e}}_{j}+\lambda_{j2}e_{j}\right]\\ = & \tau_{j}-\tau_{j,he}+M_{j}\left[\eta_{j1}\nu_{j}-\eta_{j2}{\dot{q}}_{j^{\prime }}\left(t-d_{j^{\prime }}\right)+\lambda_{j1}{\dot{e}}_{j}+\lambda_{j2}e_{j}\right]-C_{j}{\dot{q}}_{j}-G_{j}-B_{j}. \end{array}$$

where if j=m, $j^{\prime }=s$ and $\tau_{j,he}=\tau_{h}$; if j=s, $j^{\prime }=m$ and $\tau_{j,he}=-\tau_{e}$.

In addition, the differential of tracking errors $e_{m}$ and $e_{s}$ can be described as

(13) $$\begin{array}{cc} \; & {\dot{e}}_{m}\left(t\right)={\dot{q}}_{m}\left(t\right)-{\dot{q}}_{s}\left(t-d_{s}\right)\left(1-{\dot{d}}_{s}\right),\\ \; & {\dot{e}}_{s}\left(t\right)={\dot{q}}_{s}\left(t\right)-{\dot{q}}_{m}\left(t-d_{m}\right)\left(1-{\dot{d}}_{m}\right). \end{array}$$

It is obvious that the unknown time-varying delay information ${\dot{d}}_{m}$ and ${\dot{d}}_{s}$ are introduced in the ${\dot{e}}_{m}\left(t\right)$ and ${\dot{e}}_{s}\left(t\right)$. Therefore, in order to simplify the presentation and avoid introducing the unknown time delay information into the controller design, we define another form of velocity error functions $e_{mv}$ and $e_{sv}$ instead of ${\dot{e}}_{m}\left(t\right)$ and ${\dot{e}}_{s}\left(t\right)$, respectively, as

(14) $$\begin{array}{cc} \; & e_{mv}={\dot{q}}_{m}\left(t\right)-{\dot{q}}_{s}\left(t-d_{s}\right),\\ \; & e_{sv}={\dot{q}}_{s}\left(t\right)-{\dot{q}}_{m}\left(t-d_{m}\right). \end{array}$$

Then, ${\dot{e}}_{m}\left(t\right)$ and ${\dot{e}}_{s}\left(t\right)$ can be rewritten as

(15) $$\begin{array}{cc} \; & {\dot{e}}_{m}\left(t\right)=e_{mv}+{\dot{q}}_{s}\left(t-d_{s}\right){\dot{d}}_{s},\\ \; & {\dot{e}}_{s}\left(t\right)=e_{sv}+{\dot{q}}_{m}\left(t-d_{m}\right){\dot{d}}_{m}. \end{array}$$

$M_{j}{\dot{s}}_{j}$ in (12) can be rewritten as

(16) $$\begin{array}{cc} M_{j}{\dot{s}}_{j}= & \tau_{j}-\tau_{j,he}+M_{j}\left[\eta_{j1}\nu_{j}-\eta_{j2}{\dot{q}}_{j^{\prime }}\left(t-d_{j^{\prime }}\right)+\lambda_{j1}{\dot{e}}_{jv}\right.\\ \; & \;+\lambda_{j2}e_{j}+\lambda_{j1}{\dot{q}}_{j^{\prime }}\left(t-d_{j^{\prime }}\right){\dot{d}}_{j^{\prime }}]-C_{j}{\dot{q}}_{j}-G_{j}-B_{j}. \end{array}$$

With the definition of dynamic uncertainties in (2) and environment torque $\tau_{e}$, we have

(17) $$\begin{array}{cc} M_{m}{\dot{s}}_{m}= & \tau_{m}-\tau_{h}+M_{mo}\left[\eta_{m1}\nu_{m}-\eta_{m2}{\dot{q}}_{s}\left(t-d_{s}\right)\right.\\ \; & \;+\lambda_{m1}{\dot{e}}_{mv}+\lambda_{m2}e_{m}+\lambda_{m1}{\dot{q}}_{s}\left(t-d_{s}\right){\dot{d}}_{s}]\\ \; & \;-C_{mo}{\dot{q}}_{m}-G_{mo}+P_{m},\\ M_{s}{\dot{s}}_{s}= & \tau_{s}+M_{so}\left[\eta_{s1}\nu_{s}-\eta_{s2}{\dot{q}}_{m}\left(t-d_{m}\right)\right.\\ \; & \;+\lambda_{s1}{\dot{e}}_{sv}+\lambda_{s2}e_{s}+\lambda_{s1}{\dot{q}}_{m}\left(t-d_{m}\right){\dot{d}}_{m}]\\ \; & \;-C_{so}{\dot{q}}_{s}-G_{so}+P_{s}. \end{array}$$

where $P_{j}$ is the term of dynamic uncertainties, which has

$$\begin{array}{cc} P_{m}= & -\Delta M_{m}\left[\eta_{m1}\nu_{m}-\eta_{m2}{\dot{q}}_{s}\left(t-d_{s}\right)+\lambda_{m1}{\dot{e}}_{mv}\right.\\ & \;+\lambda_{m2}e_{m}+\lambda_{m1}{\dot{q}}_{s}\left(t-d_{s}\right){\dot{d}}_{s}]\\ & \;-\Delta C_{m}{\dot{q}}_{m}-\Delta G_{m}-B_{m},\\ P_{s}= & -\Delta M_{s}\left[\eta_{s1}\nu_{s}-\eta_{s2}{\dot{q}}_{m}\left(t-d_{m}\right)+\lambda_{s1}{\dot{e}}_{sv}\right.\\ & \;+\lambda_{s2}e_{s}+\lambda_{s1}{\dot{q}}_{m}\left(t-d_{m}\right){\dot{d}}_{m}]\\ & \;-\left(\Delta C_{s}+D_{e}\right){\dot{q}}_{s}-\Delta G_{s}-B_{s}-S_{e}q_{s}. \end{array}$$

It is well known that the RBF neural network can approximate the smooth nonlinear function with arbitrary precision. The $P_{j}$ can be described as

(18) $$\begin{array}{c} P_{j}=\theta_{j}^{T}Y_{j}\left(Z_{j}\right)+\epsilon_{j}. \end{array}$$

where $\theta_{j}\in R^{k\times n}$ is the ideal approximate parameter matrix of RBF neural network, $Y\left(Z_{j}\right)=\left[y_{j1}\left(Z_{j}\right),y_{j2}\left(Z_{j}\right)\right.$ $\left.,\ldots ,y_{jl}\left(Z_{j}\right)\right]\in R^{l\times 1}$ is the Gaussian basis function vector, which can be calculated by $Z_{j}$:

(19) $$\begin{array}{c} y_{ji}\left(Z_{j}\right)=e^{-\frac{{\left(Z_{j}-c_{i}\right)}^{T}\left(Z_{j}-c_{i}\right)}{2b_{j}^{2}}},i=1,2,\ldots ,l. \end{array}$$

where $c_{i}\in R^{1\times 5n}$ is the Gaussian center function vector of *i*-th hidden layer node and $b_{j}$ is the width of Gaussian function. $Z_{j}={\left[\mu_{j1}^{T},\mu_{j2}^{T},{\dot{q}}_{j^{\prime }}^{T}\left(t-d_{j^{\prime }}\right),q_{j}^{T},{\dot{q}}_{j}^{T}\right]}^{T}$. $\mu_{j1}$ and $\mu_{j2}$ are defined as

(20) $$\begin{array}{cc} \; & \mu_{j1}=-{\dot{\nu }}_{j}+\lambda_{j1}{\dot{e}}_{jv}+\lambda_{j2}e_{j},\\ \; & \mu_{j2}=s_{j}-{\dot{q}}_{j}. \end{array}$$

**Remark** **1.** *In this paper, the dynamic uncertainties are defined as a new form $P_{m}$ and $P_{s}$, so that the acceleration signals cannot be utilized for parameter estimation. We first give the Lyapunov function as $V_{1}=\frac{1}{2}s_{m}^{T}M_{m}s_{m}+\frac{1}{2}s_{s}^{T}M_{s}s_{s}$, and then define the uncertain parts in ${\dot{V}}_{1}$ as the dynamic uncertainties to estimate. However, in most of the existing work, $\Delta M_{j}{\ddot{q}}_{j}$, $\Delta C_{j}{\dot{q}}_{j}$, and $\Delta G_{j}$ are directly considered as the uncertain part to compensate, which means the acceleration signal has to be introduced to obtain the parameters of $\Delta M_{j}$.*

**Figure 1:**
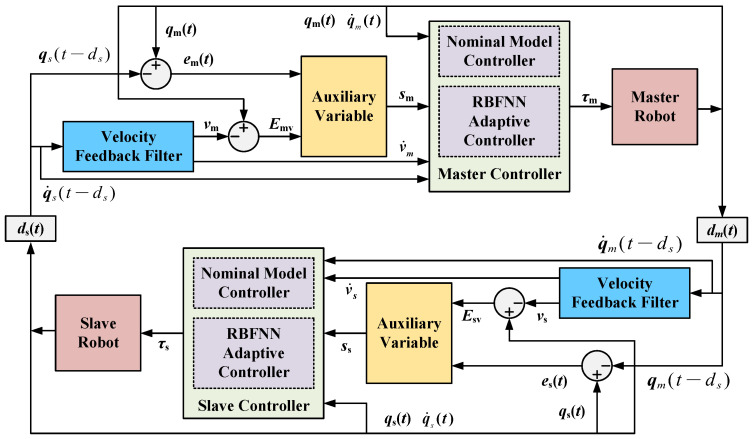
Proposed control structure.

Then, the adaptive control scheme of the teleoperation system with asymmetric time-varying delays and dynamic uncertainties is shown in Figure 1.

The control laws are proposed as

(21) $$\begin{array}{cc} \tau_{j}= & \tau_{j1}+\tau_{j2}. \end{array}$$

where $\tau_{j1}$ and $\tau_{j2}$ are designed as

(22) $$\begin{array}{cc} \tau_{j1}= & -M_{jo}\left[\eta_{j1}\nu_{j}-\eta_{j2}{\dot{q}}_{j^{\prime }}\left(t-d_{j^{\prime }}\right)+\lambda_{j1}{\dot{e}}_{jv}\right.\\ \; & \left.\;+\lambda_{j2}e_{j}\right]-C_{jo}\left(s_{j}-{\dot{q}}_{j}\right)+G_{jo},\\ \tau_{j2}= & -{\hat{\theta }}_{j}^{T}Y_{j}\left(Z_{j}\right)-\frac{{\hat{D}}_{j^{\prime }}\mu_{j3}^{T}\mu_{j3}s_{j}}{2a_{j1}^{2}}-\frac{{\hat{\omega }}_{j}s_{j}}{2a_{j2}^{2}}-K_{j}s_{j}. \end{array}$$

where $\mu_{j3}=\lambda_{j1}M_{jo}{\dot{q}}_{j^{\prime }}\left(t-d_{j^{\prime }}\right)$; the adaptive laws are presented as

(23) $$\begin{array}{cc} \; & {\dot{\hat{\theta }}}_{j}=\Lambda_{j1}Y_{j}\left(Z_{j}\right)s_{j}^{T}-{\hat{\theta }}_{j},\\ \; & {\dot{\hat{D}}}_{j^{\prime }}=\frac{\Lambda_{j2}}{2a_{j1}^{2}}{\left(s_{j}^{T}\mu_{j3}\right)}^{2}-{\hat{D}}_{j^{\prime }},\\ \; & {\dot{\hat{\omega }}}_{j}=\frac{\Lambda_{j3}}{2a_{j2}^{2}}s_{j}^{T}s_{j}-{\hat{\omega }}_{j}. \end{array}$$

**Remark** **2.** *Different from the assumption of the time delay derivation ${\dot{d}}_{j}$ in [17,25], the bounds of ${\dot{d}}_{j}$ need not be known and set as ${\dot{d}}_{j}<1$ in our proposed control scheme. In control laws, ${\dot{e}}_{jv}$ in (14) is applied instead of ${\dot{e}}_{j}$, which means the ${\dot{d}}_{j}$ does not need to be used. We design the adaptive parameter ${\hat{D}}_{j}$ to compensate this replacement.*

## 4. Stability Analysis

**Theorem** **1.** *With the velocity feedback filter (6), auxiliary variable (9), proposed control laws (21), (22) and adaptive laws (23), if Assumptions 1 and 2 hold, the parameters satisfy that $K_{j}$, $\eta_{j1}$, $\eta_{j2}$, $\lambda_{j1}$, $\lambda_{j2}$, and $\Lambda_{j1}$ are all positive definite diagonal matrices; and $\Lambda_{j1}$ and $\Lambda_{j3}$ are all positive constants, for j=m,s. Then, the closed-loop teleoperation system under time-varying delays and model uncertainties is asymptotically stable.*

**Proof.**  We design the Lyapunov–Krasovskii function *V* as

(24) $$\begin{array}{c} V=V_{1}+V_{2}+V_{3}. \end{array}$$

where for j=m,s, $j^{\prime }=s,m$, we have

(25) $$\begin{array}{c} V_{1}=\sum_{m,s}\frac{1}{2}s_{j}^{T}M_{j}s_{j}. \end{array}$$

(26) $$\begin{array}{c} V_{2}=\sum_{m,s}\left[\frac{1}{2}\mathrm{tr}\left({\tilde{\theta }}_{j}^{T}\Lambda_{j1}^{-1}{\tilde{\theta }}_{j}\right)+\frac{\Lambda_{j2}^{-1}{\tilde{D}}_{j^{\prime }}^{2}}{2}+\frac{\Lambda_{j3}^{-1}{\tilde{\omega }}_{j}^{2}}{2}\right]. \end{array}$$

(27) $$\begin{array}{c} V_{3}=\sum_{m,s}L_{j}\int_{t-{\bar{d}}_{j}}^{t}\frac{{\bar{d}}_{j}-t+\beta }{{\bar{d}}_{j}}s_{j}^{T}s_{j}d\beta . \end{array}$$

where $\tilde{\theta }=\theta -\hat{\theta }$, ${\tilde{D}}_{j^{\prime }}=D_{j^{\prime }}-{\hat{D}}_{j^{\prime }}$, ${\tilde{\omega }}_{j}=\omega_{j}-{\hat{\omega }}_{j}$, and $L_{j}\geq 0$ is a known constant.

First, the time derivative of $V_{1}$ can be described as

(28) $$\begin{array}{cc} \; & {\dot{V}}_{1}=\sum_{m,s}\left(s_{j}^{T}M_{j}{\dot{s}}_{j}+\frac{1}{2}s_{j}^{T}{\dot{M}}_{j}s_{j}\right). \end{array}$$

Based on Property 2 of dynamic (1) and the description of (17), we can obtain that

(29) $$\begin{array}{cc} {\dot{V}}_{1}= & \sum_{m,s}s_{j}^{T}\left(M_{j}{\dot{s}}_{j}+C_{j}s_{j}\right)\\ = & \sum_{m,s}s_{j}^{T}\left\{\tau_{j}-\tau_{j,he}+M_{jo}\left[\eta_{j1}\nu_{j}-\eta_{j2}{\dot{q}}_{j^{\prime }}\left(t-d_{j^{\prime }}\right)+\lambda_{j1}{\dot{e}}_{jv}\right.\right.\\ \; & \;\left.\left.+\lambda_{j2}e_{j}\right]+\lambda_{j1}M_{j}{\dot{q}}_{j^{\prime }}\left(t-d_{j^{\prime }}\right){\dot{d}}_{j^{\prime }}+C_{jo}\left(s_{j}-{\dot{q}}_{j}\right)-G_{jo}+P_{j}\right\} \end{array}$$

With the control laws in (22) and the RBF neural network description of $P_{j}$ in (18), we have

(30) $$\begin{array}{cc} {\dot{V}}_{1}= & \sum_{m,s}\left\{s_{j}^{T}\left[{\tilde{\theta }}_{j}^{T}Y_{j}\left(Z_{j}\right)-\frac{{\hat{D}}_{j^{\prime }}\mu_{j3}^{T}\mu_{j3}s_{j}}{2a_{j1}^{2}}-\frac{{\hat{\omega }}_{j}s_{j}}{2a_{j2}^{2}}\right]-s_{j}^{T}K_{j}s_{j}\right.\\ \; & \;+s_{j}^{T}\lambda_{j1}M_{j}{\dot{q}}_{j^{\prime }}\left(t-d_{j^{\prime }}\right){\dot{d}}_{j^{\prime }}+s_{j}^{T}\left(\epsilon_{j}-\tau_{j,he}\right)\}\\ \leq & \sum_{m,s}\left\{s_{j}^{T}\left[{\tilde{\theta }}_{j}^{T}Y_{j}\left(Z_{j}\right)-\frac{{\hat{D}}_{j^{\prime }}\mu_{j3}^{T}\mu_{j3}s_{j}}{2a_{j1}^{2}}-\frac{{\hat{\omega }}_{j}s_{j}}{2a_{j2}^{2}}\right]-s_{j}^{T}K_{j}s_{j}\right.\\ \; & \left.\;+\frac{|{\dot{d}}_{j^{\prime }}|}{2a_{j1}^{2}}s_{j}^{T}\mu_{j3}^{T}\mu_{j3}s_{j}+\frac{∥\epsilon_{j}-\tau_{j,he}{∥}^{2}}{2a_{j2}^{2}}s_{j}^{T}s_{j}+\frac{a_{j1}^{2}+a_{j2}^{2}}{2}\right\}. \end{array}$$

As $|{\dot{d}}_{j^{\prime }}|\leq D_{j^{\prime }}$ and $∥\epsilon_{j}-\tau_{j,he}{∥}^{2}\leq \omega_{j}$, we can obtain

(31) $$\begin{array}{cc} {\dot{V}}_{1}\leq & \sum_{m,s}\left\{s_{j}^{T}{\tilde{\theta }}_{j}^{T}Y_{j}\left(Z_{j}\right)+\frac{{\tilde{D}}_{j^{\prime }}}{2a_{j1}^{2}}s_{j}^{T}\mu_{j3}^{T}\mu_{j3}s_{j}+\frac{{\tilde{\omega }}_{j}}{2a_{j2}^{2}}s_{j}^{T}s_{j}-s_{j}^{T}K_{j}s_{j}+\frac{a_{j1}^{2}+a_{j2}^{2}}{2}\right\}. \end{array}$$

The time derivative of $V_{2}$ can be calculated as

(32) $$\begin{array}{c} V_{2}=\sum_{m,s}\left[\mathrm{tr}\left({\tilde{\theta }}_{j}^{T}\Lambda_{j1}^{-1}{\dot{\tilde{\theta }}}_{j}\right)+\Lambda_{j2}^{-1}{\tilde{D}}_{j^{\prime }}{\dot{\tilde{D}}}_{j^{\prime }}+\Lambda_{j3}^{-1}{\tilde{\omega }}_{j}{\dot{\tilde{\omega }}}_{j}\right] \end{array}$$

With the definition of ${\tilde{\theta }}_{j}$, ${\tilde{D}}_{j^{\prime }}$, ${\tilde{\omega }}_{j}$, and the adaptive laws in (23), ${\dot{V}}_{2}$ can be written as

(33) $$\begin{array}{cc} {\dot{V}}_{2}= & \sum_{m,s}\left\{\mathrm{tr}\left[{\tilde{\theta }}_{j}^{T}Y_{j}\left(Z_{j}\right)s_{j}^{T}\right]-\frac{{\tilde{D}}_{j^{\prime }}}{2a_{j1}^{2}}{\left(s_{j}^{T}\mu_{j3}\right)}^{2}-\frac{{\tilde{\omega }}_{j}}{2a_{j2}^{2}}s_{j}^{T}s_{j}\right.\\ \; & \;\left.+\mathrm{tr}\left({\tilde{\theta }}_{j}^{T}\Lambda_{j1}^{-1}{\hat{\theta }}_{j}\right)+\Lambda_{j2}^{-1}{\tilde{D}}_{j^{\prime }}{\hat{D}}_{j^{\prime }}+\Lambda_{j3}^{-1}{\tilde{\omega }}_{j}{\hat{\omega }}_{j}\right\}. \end{array}$$

For $\mathrm{tr}\left({\tilde{\theta }}_{j}^{T}\Lambda_{j1}^{-1}{\hat{\theta }}_{j}\right)$, $\Lambda_{j2}^{-1}{\tilde{D}}_{j}{\hat{D}}_{j}$, and $\Lambda_{j3}^{-1}{\tilde{\omega }}_{j}\hat{\omega }$, the following inequalities are always established:

(34) $$\begin{array}{cc} \; & \mathrm{tr}\left({\tilde{\theta }}_{j}^{T}\Lambda_{j1}^{-1}{\hat{\theta }}_{j}\right)\leq -\frac{1}{2}\mathrm{tr}\left({\tilde{\theta }}_{j}^{T}\Lambda_{j1}^{-1}{\tilde{\theta }}_{j}\right)+\frac{1}{2}\mathrm{tr}\left(\theta_{j}^{T}\Lambda_{j1}^{-1}\theta_{j}\right),\\ \; & \Lambda_{j2}^{-1}{\tilde{D}}_{j}{\hat{D}}_{j}\leq -\frac{\Lambda_{j2}^{-1}}{2}{\tilde{D}}_{j}^{2}+\frac{\Lambda_{j2}^{-1}}{2}D_{j}^{2},\\ \; & \Lambda_{j3}^{-1}{\tilde{\omega }}_{j}\hat{\omega }\leq -\frac{\Lambda_{j3}^{-1}}{2}{\tilde{\omega }}^{2}+\frac{\Lambda_{j3}^{-1}}{2}\omega^{2}. \end{array}$$

Then, we can obtain

(35) $$\begin{array}{cc} {\dot{V}}_{2}= & \sum_{m,s}\left\{\mathrm{tr}\left[{\tilde{\theta }}_{j}^{T}Y_{j}\left(Z_{j}\right)s_{j}^{T}\right]-\frac{{\tilde{D}}_{j^{\prime }}}{2a_{j1}^{2}}{\left(s_{j}^{T}\mu_{j3}\right)}^{2}\frac{{\tilde{\omega }}_{j}}{2a_{j2}^{2}}s_{j}^{T}s_{j}\right.\\ \; & \;\left.-\frac{1}{2}\mathrm{tr}\left({\tilde{\theta }}_{j}^{T}\Lambda_{j1}^{-1}{\tilde{\theta }}_{j}\right)-\frac{1}{2}\Lambda_{j2}^{-1}{\tilde{D}}_{j^{\prime }}^{2}-\frac{1}{2}\Lambda_{j3}^{-1}{\tilde{\omega }}_{j}^{2}+\Psi_{j}\right\}. \end{array}$$

where $\Psi_{j}=\frac{1}{2}\mathrm{tr}\left(\theta_{j}^{T}\Lambda_{j1}^{-1}\theta_{j}\right)+\frac{\Lambda_{j2}^{-1}}{2}D_{j}^{2}+\frac{\Lambda_{j3}^{-1}}{2}\omega^{2}$ is the positive constant.

For $V_{3}$, we have

(36) $$\begin{array}{cc} {\dot{V}}_{3}= & \sum_{j=m,s}\left[L_{j}s_{j}^{T}s_{j}-\frac{L_{j}}{{\bar{d}}_{j}}\int_{t-{\bar{d}}_{j}}^{t}s_{j}^{T}\left(\beta \right)s_{j}\left(\beta \right)\;d\beta \right] \end{array}$$

In addition, it is clear that $\int_{t-{\bar{d}}_{j}}^{t}\frac{{\bar{d}}_{j}-t+\beta }{{\bar{d}}_{j}}s_{j}^{T}\left(\beta \right)s_{j}\left(\beta \right)\;d\beta \leq \int_{t-{\bar{d}}_{j}}^{t}s_{j}^{T}\left(\beta \right)s_{j}\left(\beta \right)\;d\beta$. Then, we can obtain

(37) $$\begin{array}{cc} {\dot{V}}_{3}\leq & \sum_{j=m,s}\left[L_{j}s_{j}^{T}s_{j}-\frac{L_{j}}{{\bar{d}}_{j}}\int_{t-{\bar{d}}_{j}}^{t}\frac{{\bar{d}}_{j}-t+\beta }{{\bar{d}}_{j}}s_{j}^{T}\left(\beta \right)s_{j}\left(\beta \right)\;d\beta \right]. \end{array}$$

Then, combining the ${\dot{V}}_{1}$ in (31), ${\dot{V}}_{2}$ in (35), and ${\dot{V}}_{3}$ in (37), we have

(38) $$\begin{array}{cc} \dot{V}\leq & \sum_{j=m,s}\left[-s_{j}^{T}K_{j}s_{j}+s_{j}^{T}L_{j}Is_{j}-\frac{1}{2}\mathrm{tr}\left({\tilde{\theta }}_{j}^{T}\Lambda_{j1}^{-1}{\tilde{\theta }}_{j}\right)-\frac{1}{2}\Lambda_{j2}^{-1}{\tilde{D}}_{j^{\prime }}^{2}\right.\\ \; & \;\left.-\frac{1}{2}\Lambda_{j3}^{-1}{\tilde{\omega }}_{j}^{2}-\frac{L_{j}}{{\bar{d}}_{j}}\int_{t-{\bar{d}}_{j}}^{t}\frac{{\bar{d}}_{j}-t+\beta }{{\bar{d}}_{j}}s_{j}^{T}\left(\beta \right)s_{j}\left(\beta \right)\;d\beta +\frac{a_{j1}^{2}+a_{j2}^{2}}{2}+\Psi_{j}\right]\\ \leq & -\Upsilon V+\Psi . \end{array}$$

where for j=m,s, Υ and Ψ are defined as

$$\begin{array}{cc} & \Upsilon =\min\left[\frac{2\lambda_{\min}\left(K_{j}-L_{j}I\right)}{\lambda_{\max}\left(M_{j}\right)},1,\frac{1}{{\bar{d}}_{j}}\right],\\ & \Psi =\Psi_{m}+\Psi_{s}+\frac{a_{m1}^{2}+a_{m2}^{2}}{2}+\frac{a_{s1}^{2}+a_{s2}^{2}}{2}. \end{array}$$

Based on (38), it implies that

(39) $$\begin{array}{cc} 0\leq V(t)\leq & \left[V\left(0\right)-\frac{\Psi }{\Upsilon }\right]e^{-\Upsilon t}+\frac{\Psi }{\Upsilon }. \end{array}$$

We can determine that *V* is radically bounded. In addition, $s_{j}$, ${\tilde{\theta }}_{j}$, ${\tilde{D}}_{j}$, and ${\tilde{\omega }}_{j}$ are also bounded. The stability analysis mentioned above shows that the closed-loop system is asymptotically stable and the signals are bounded.

This completes the proof. □

**Remark** **3.** *In this paper, the error integrator is utilized for control scheme development. Based on the mathematical proof above, it is noted that auxiliary variable $s_{j}$ is bounded. Therefore, $\int_{0}^{t}e_{j}d\beta$ is also bounded, which implies that error $e_{j}$ needs to converge to 0. In some research works, the auxiliary variable is only designed with error and error differential, and the bounded $s_{j}$ can only obtain the bounded $e_{j}$. Thus, the proposed control scheme can improve position tracking accuracy.*

## 5. Simulation and Experimental Analysis

### 5.1. Simulation Analysis

**Figure 2:**
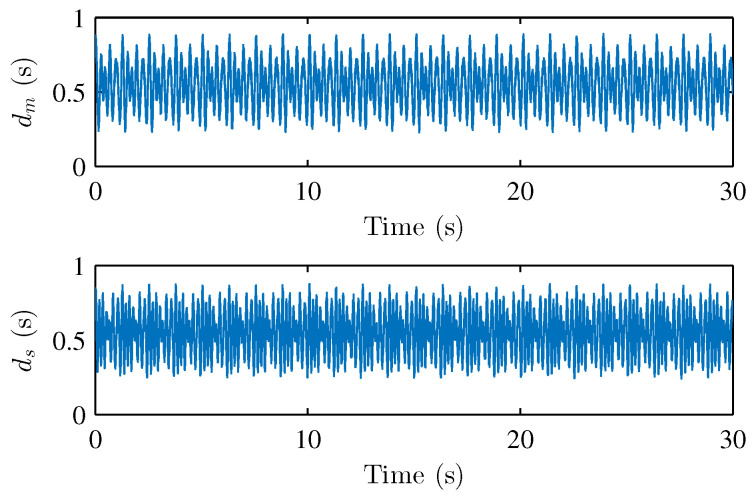
Forward and backward time-varying delays $d_{m}$ and $d_{s}$.

**Figure 3:**
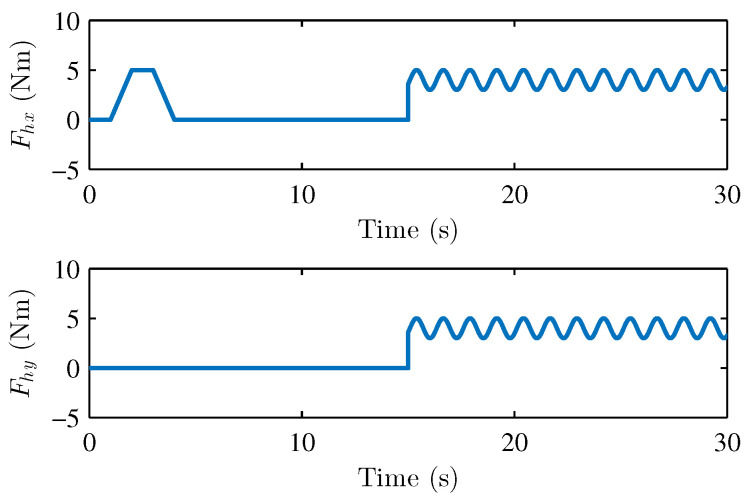
External operator torques $F_{hx}$ in x-direction and $F_{hy}$ y-direction.

The teleoperation system used for simulation consists of two robot manipulators with 2-Dof revolute joints. The asymmetric time-varying delays, uncertain dynamics, and external operator/environment forces are also considered in the simulation experiments. The dynamics of the local robot and remote robot are the same as (1), for which the mass inertia matrix $M_{j}$, centripetal Coriolis matrix $C_{j}$, gravitational torque $G_{j}$, and friction torque $B_{j}$ are defined as [39]

$$\begin{array}{cc} M_{j} & =\left[\begin{array}{cc} m_{j11} & m_{j12}\\ m_{j21} & m_{j22} \end{array}\right],\\ C_{j} & =\left[\begin{array}{cc} c_{j11} & c_{j12}\\ c_{j21} & c_{j22} \end{array}\right],\\ G_{j} & ={\left[g_{j1},g_{j2}\right]}^{T},\\ B_{j} & ={\left[b_{j1},b_{j2}\right]}^{T}. \end{array}$$

$$\begin{array}{cc} m_{j11} & =l_{j1}^{2}\left(m_{j1}+m_{j2}\right)+l_{j2}m_{j2}\left(2l_{j1}\cos q_{j2}+l_{j2}\right),\\ m_{j12} & =m_{j21}=l_{j2}^{2}m_{j2}+l_{j1}l_{j2}m_{j2}\cos q_{j2},\\ m_{22} & =l_{j2}^{2}m_{j2},\\ c_{11} & =-l_{j1}l_{j2}m_{j2}\sin q_{j2}{\dot{\dot{q}}}_{j2},\\ c_{12} & =-l_{j1}l_{j2}m_{j2}\sin q_{j2}\left({\dot{q}}_{j1}+{\dot{q}}_{j2}\right),\\ c_{21} & =l_{j1}l_{j2}m_{j2}\sin q_{j2},\\ c_{22} & =0,\\ g_{j1} & =\left(m_{j1}+m_{j2}\right)l_{j1}g\cos\left(q_{j1}\right)+m_{j2}l_{j2}g\cos\left(q_{j1}+q_{j2}\right),\\ g_{j2} & =m_{j2}l_{j2}g\cos\left(q_{j1}+q_{j2}\right),\\ b_{j1} & =k_{j1}{\dot{q}}_{j1}+k_{j2}\mathrm{sign}\left({\dot{q}}_{j1}\right),\\ b_{j2} & =k_{j3}{\dot{q}}_{j2}+k_{j4}\mathrm{sign}\left({\dot{q}}_{j2}\right). \end{array}$$

where the parameters of master and slave robots are chosen as follows: $m_{m1}=m_{s1}=3.5\;\mathrm{kg}$, $m_{m2}=m_{s2}=2.5\;\mathrm{kg}$, $l_{m1}=l_{s1}=0.3\;m$, $l_{m2}=l_{s2}=0.35\;m$, $k_{m1}=k_{m3}=0.5$, $k_{m2}=k_{m4}=0.2$, $k_{s1}=k_{s2}=k_{s3}=k_{s4}=0.3$, $g=9.8\;{m/s}^{2}$. The environment model is the same as the description in Section 2. The damping matrice is defined as $D_{e}=\mathrm{diag}\left(0.5,0.5\right)$; the spring matrice is set as $S_{e}=\mathrm{diag}\left(10.0,10.0\right)$. The asymmetric time-varying delays at the master and slave side are shown in Figure 2. The operator torques in x-direction and y-direction are assumed as shown in Figure 3.

In simulation experiments, the model perturbation parts $\Delta M_{j}$, $\Delta C_{j}$, and $\Delta G_{j}$ are introduced into the dynamic models of the master and slave robot; they can be presented as $\Delta M_{j}=0.1\sin\left(10t\right)M_{j}$, $\Delta C_{j}=0.1\sin\left(10t\right)C_{j}$, and $\Delta G_{j}=0.1\sin\left(5t\right)G_{j}$, respectively. In addition, the parameters of nominal models are set as $m_{mo1}=m_{s1}=2\;\mathrm{kg}$, $m_{mo2}=m_{s2}=1\;\mathrm{kg}$, $l_{m1}=l_{s1}=0.3\;m$, $l_{m2}=l_{s2}=0.3\;m$. We consider simulating the uncertain conditions of teleoperation dynamics by adding the model perturbation parts and defining the inaccurate nominal model parameters, which are employed to verify the effectiveness of our proposed methods. The controller parameters are set as follows: in master controller—$\eta_{m1}=\mathrm{diag}\left(5.0,5.0\right)$, $\eta_{m2}=\mathrm{diag}\left(1.0,1.0\right)$, $\lambda_{m1}=\mathrm{diag}\left(2.0,2.0\right)$, $\lambda_{m2}=\mathrm{diag}\left(1.5,1.5\right)$, $K_{m}=\mathrm{diag}\left(25.0,25.0\right)$, $a_{m1}=a_{m2}=1$, $\Lambda_{m1}=\mathrm{diag}\left(0.9,0.9\right)$, $\Lambda_{m2}=\Lambda_{m3}=0.9$; in slave controller—$\eta_{s1}=\mathrm{diag}\left(5.0,5.0\right)$, $\eta_{s2}=\mathrm{diag}\left(1.0,1.0\right)$, $\lambda_{s1}=\mathrm{diag}\left(2.0,2.0\right)$, $\lambda_{s2}=\mathrm{diag}\left(2.5,2.5\right)$, $K_{s}=\mathrm{diag}\left(25.0,25.0\right)$, $a_{s1}=a_{s2}=1$, $\Lambda_{s1}=\mathrm{diag}\left(0.9,0.9\right)$, $\Lambda_{s2}=\Lambda_{s3}=0.9$.

**Figure 4:**
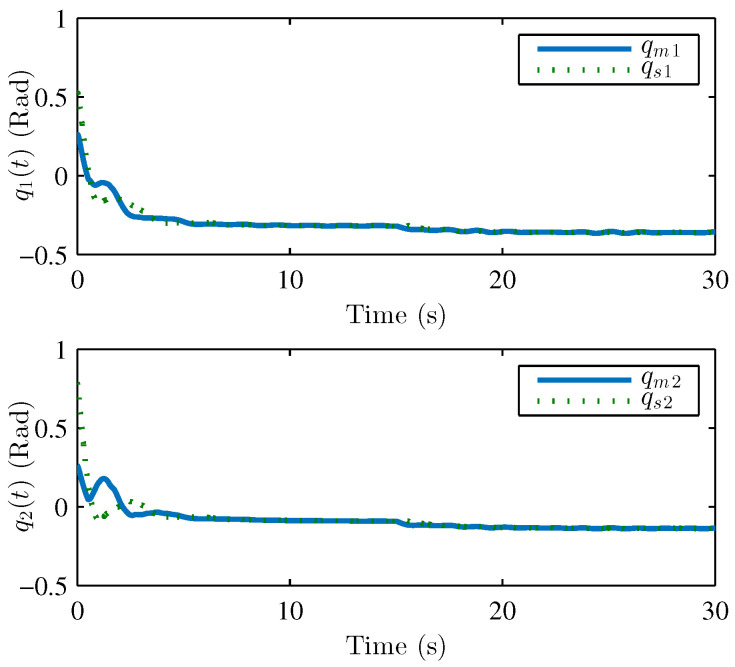
Joint positions of master robot $q_{m1},q_{m2}$ and slave robot $q_{s1},q_{s2}$.

**Figure 5:**
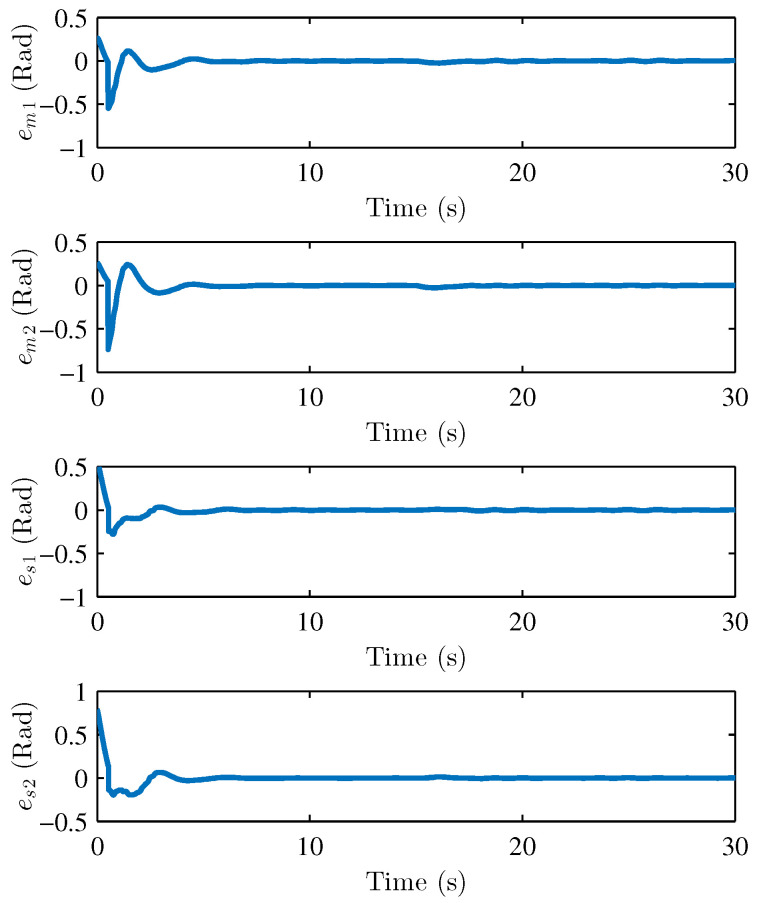
Position tracking errors at master side $e_{m1},e_{m2}$ and slave side $e_{s1},e_{s2}$.

**Figure 6:**
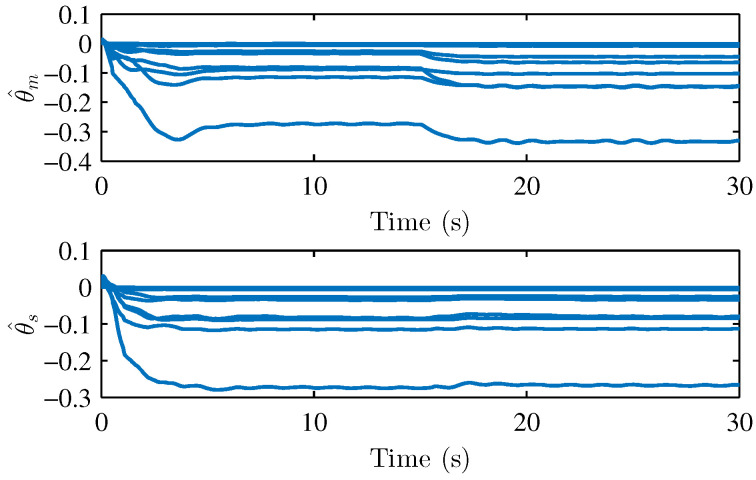
Adaptive parameters $\theta_{m}$ and $\theta_{s}$ in RBF neural network for uncertain parts estimation.

**Figure 7:**
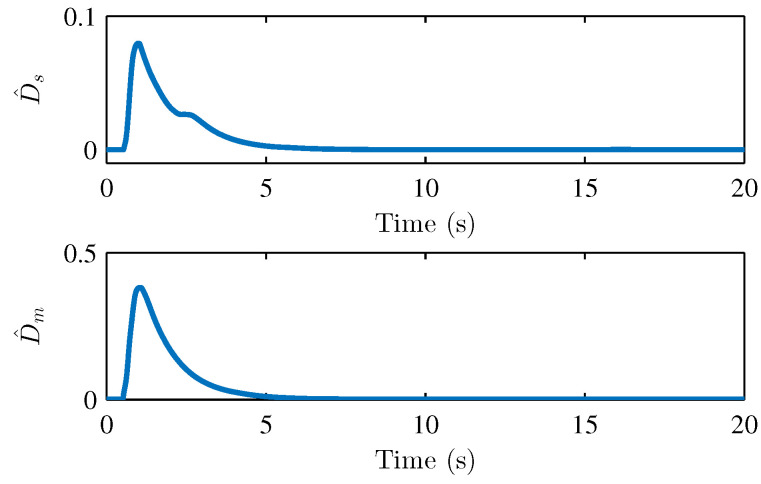
Adaptive parameters ${\hat{D}}_{s}$ and ${\hat{D}}_{m}$.

**Figure 8:**
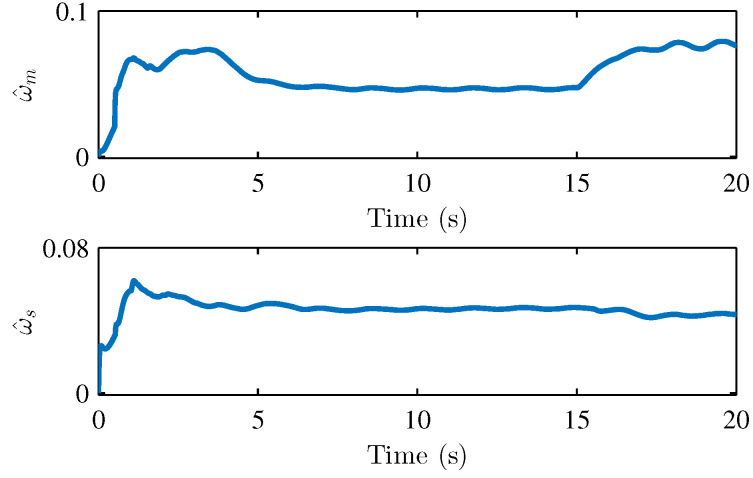
Adaptive parameters ${\hat{\omega }}_{m}$ and ${\hat{\omega }}_{s}$.

We performed two parts of the simulation experiment to verify the control effectiveness of the developed control scheme. First, The simulation experiments are performed to show the stability and performances of the teleoperation system and verify Theorem 1. Based on the simulation conditions and parameters set above, the results of the simulation are shown in Figure 4 and Figure 5, respectively. Figure 4 presents the joint position of the master robot and the slave robot. Figure 5 shows the position tracking errors between the master and the slave robot. It can be seen that the joint positions of master and slave robots achieve good tracking performances. The joint positions are changed when the active operating forces are exerted on the master robot, and the position tracking errors can still converge to 0. Figure 5 also illustrates that the tracking errors $e_{m}$ and $e_{s}$ are bounded. Figure 6, Figure 7 and Figure 8 show the adaptive parameters of ${\hat{\theta }}_{m}$, ${\hat{\theta }}_{s}$, ${\hat{D}}_{m}$, ${\hat{D}}_{s}$, ${\hat{\omega }}_{m}$, and ${\hat{\omega }}_{s}$. It is shown that these adaptive parameters are all bounded.

**Figure 9:**
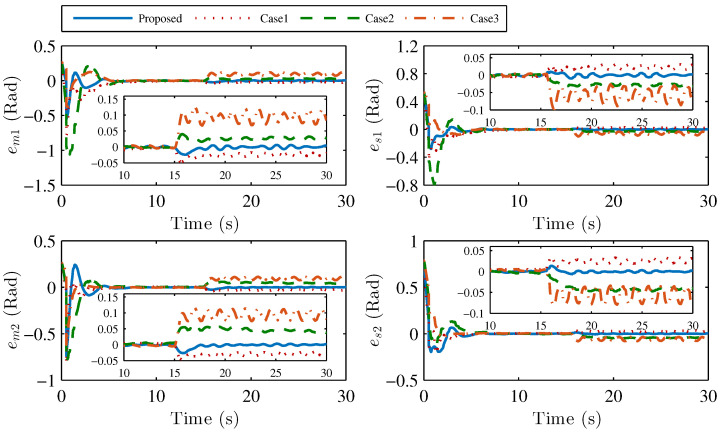
Comparisons of position tracking with different control methods: our proposed method, simulation case 1, simulation case 2, and simulation case 3.

Second, the simulation comparisons with the other three control methods are presented. In this part, two adaptive control algorithms in [21,23] and an improved P + d control scheme in [12] are introduced to illustrate the performance of our method. In the works [21,23], the RBF neural network is implied to estimate the uncertainties. These two control schemes have good control performance. However, compared with our method, on the one hand, the acceleration signals are used in [21,23]; on the other hand, the integral of position error is not considered in the controller design, which may make the system have steady-state error under the action of different operator forces. Figure 9 shows the control effect comparison between our proposed method and other control methods, where simulation case 1, simulation case 2, and simulation case 3 represent the position tracking error curves using the control methods in the works [21,23], and [12], respectively. As it can be seen, the position tracking error of these control methods can converge to a small area of 0 with a short-time operator force. When the continuous operating forces are applied to the master robot, there are large tracking errors with the control method in simulation case 1, simulation case 2, and simulation case 3.

In this paper, the Root Mean Square Error (RMSE) of position tracking errors is employed to evaluate the control effect under different methods. The RMSE can be defined as

(40) $$\begin{array}{c} RMSE=\sqrt{\frac{1}{n}\sum_{i=1}^{n}{\left(e_{i}\right)}^{2}} \end{array}$$

where $e_{i}$ represents the *i*-th error in the error sequence, and *n* represents the length of the error sequence. In fact, the RMSE index can be introduced to characterize the degree of position tracking error distribution of master and slave robots. The smaller the RMSE is, the closer the tracking error distribution between the master robot and the slave robot is, which means that the better the tracking control performance is.

The RMSEs of the position tracking errors with these three control schemes are shown in Table 1. In Table 1, the minimum RMSE value in each row is shown in bold. It can be seen from these data that the position tracking errors with the proposed control method have smaller RMSE values, which means that the master and slave robots have better position tracking performance based on the proposed control method. It also can be found that with the action of nonzero external environmental forces, larger steady-state errors are introduced into position tracking errors of the master and slave robots based on method 1, method 2, and method 3. Therefore, the tracking error RMSE values based on these three methods are relatively large. This result also verifies the validity of the auxiliary function constructed by the error integral term in this proposed method.

### 5.2. Experiment Analysis

**Figure 10:**
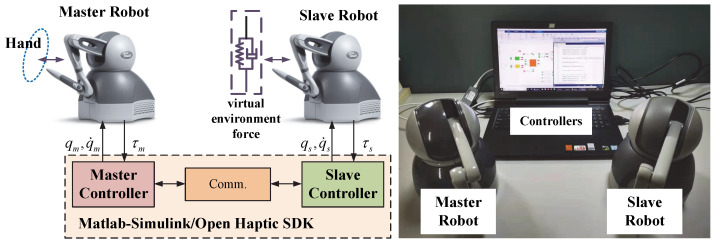
Experimental system used in our work, the left part is the system structure and the right side is the actual experiment system.

In this part, the experiments are performed to verify the validity of proposed control method. The experiment system is composed of two Phantom Omni devices with actuated 3-DOF. One Phantom Omni is denoted as the master robot and another one is denoted as the slave robot. These two devices run in the MATLAB/Simulink environment based on OpenHaptic SDK and S-Function. In the experiment, the first three active joints of these two robots are used, and the passive joints are fixed by ropes to prevent the influence of their rotation. The dynamic descriptions and the parameters of the devices are obtained from [40]. The parameters of the virtual environment force model are defined as follows: $D_{e}=\mathrm{diag}$(0.01, 0.01, 0.01) and $S_{e}=\mathrm{diag}$(0.1, 0.1, 0.1). The parameters of the master controller and slave controller are set as follows: in master controller—$\eta_{m1}=\eta_{m2}=\mathrm{diag}$(15.0, 15.0, 15.0), $\lambda_{m1}=\mathrm{diag}\left(20.0,20.0,20.0\right)$, $\lambda_{m2}=\mathrm{diag}\left(15.0,20.0,20.0\right)$, $K_{m}=\mathrm{diag}\left(25.0,20.0,30.0\right)$, $a_{m1}=a_{m2}=5$, $\Lambda_{m1}=0.9I_{15\times 15}$, $\Lambda_{m2}=\Lambda_{m3}=0.9$; in slave controller—$\eta_{s1}=\eta_{s2}=\mathrm{diag}$(10.0, 10.0, 10.0), $\lambda_{s1}=\mathrm{diag}$(20.0, 23.0, 24.0), $\lambda_{s2}=\mathrm{diag}(25.0,\;$22.0,25.0), $K_{s}=\mathrm{diag}$(40.0, 35.0, 30.0), $a_{s1}=a_{s2}=5$, $\Lambda_{s1}=0.9I_{15\times 15}$, $\Lambda_{s2}=\Lambda_{s3}=0.9$. In order to reflect the control performance of the closed-loop teleoperation system, we have added a spring-damped model to simulate the action of the slave Phantom Omni devices on the environmental forces. In the experiment, the operator drags the end of the master robot to achieve an approximate circular periodic trajectory and feels the force from the slave robot. The slave robot moves along the trajectory of the master robot with the force action of the virtual spring-damped model. The schematic diagram and the structure of the environment system are shown in Figure 10.

**Figure 11:**
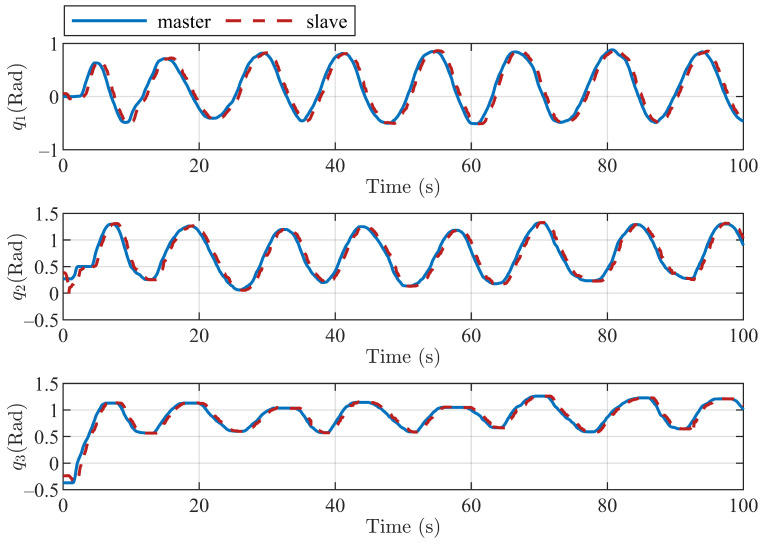
Tracking control performance of three active joints of master and slave robots based on the proposed algorithm.

**Figure 12:**
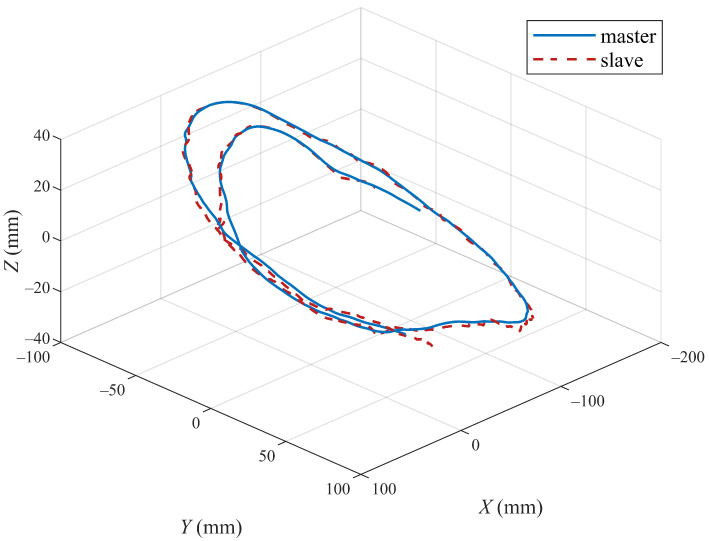
Tracking control performance of end-effectors of master and slave robots based on the proposed algorithm.

The position tracking performances of the three joints and end position of the master and slave robots with the proposed method are shown in Figure 11 and Figure 12. Figure 11 shows the tracking of the three active joints of the master and slave robots under the action of the operator dragging and the virtual environment force Figure 12 shows the trajectory tracking of the master and slave robots under the above experimental conditions in the Cartesian coordinate frame. We only intercepted part of the trajectory curves to better show the trajectory tracking performances of the robot end-effectors.

**Figure 13:**
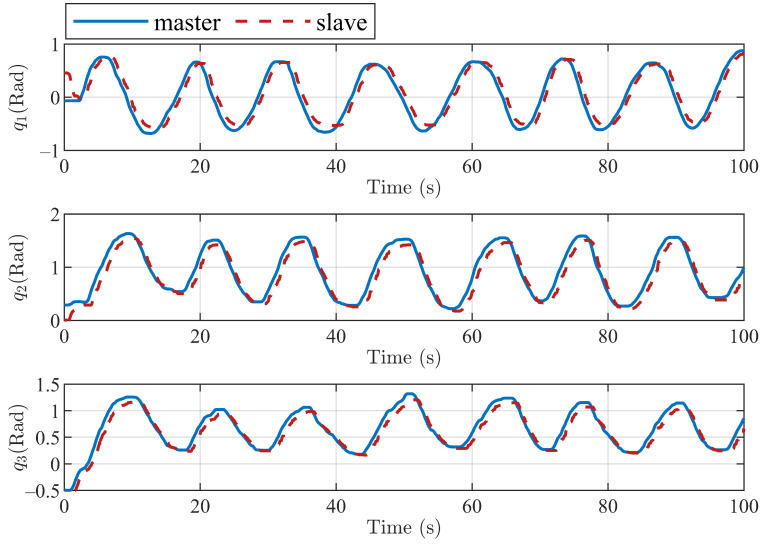
Tracking control performance of three active joints of master and slave robots under experiment case 1.

**Figure 14:**
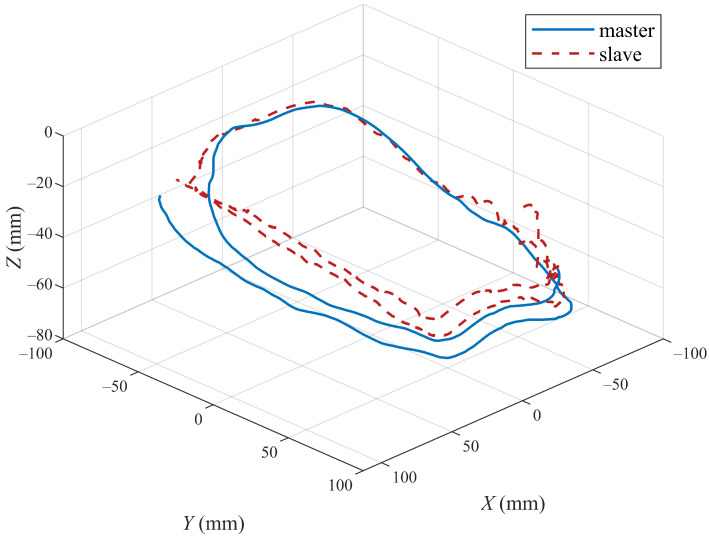
Tracking control performance of end-effectors of master and slave robots under experiment case 1.

**Figure 15:**
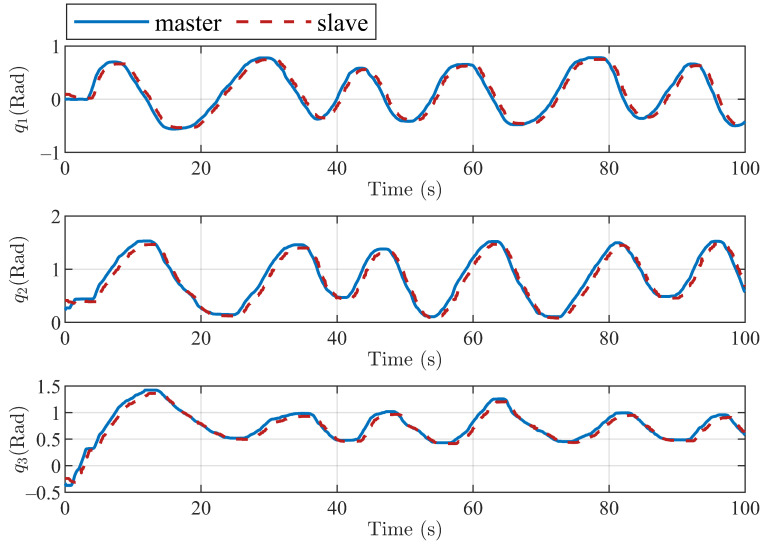
Tracking control performance of three active joints of master and slave robots under experiment case 2.

**Figure 16:**
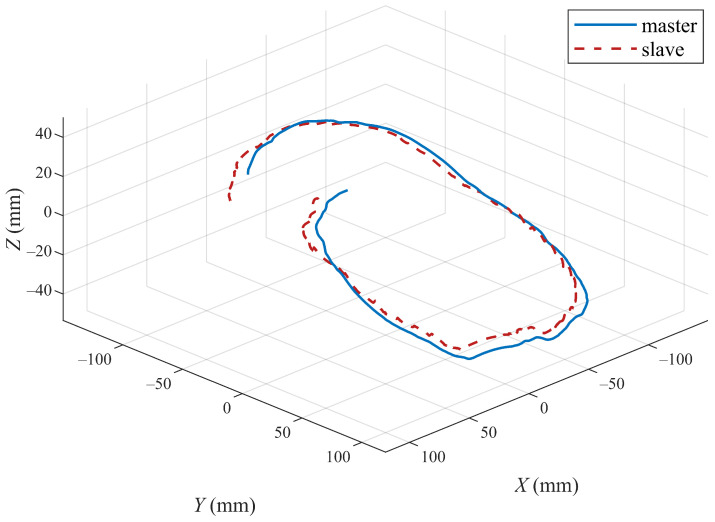
Tracking control performance of end-effectors of master and slave robots under experiment case 2.

In addition, to better verify the efficiency of the proposed method, the trajectory tracking performances of the master and slave robots using the control algorithms in [12,23] are also presented in Figure 13, Figure 14, Figure 15 and Figure 16. For the convenience of analysis and expression, we define the experimental results based on the method in [12] as experiment case 1, and the results based on the method in [23] as experiment case 2. The conditions of these two experiments are the same as the previous experiment. Figure 13 and Figure 14 show the tracking of the three active joints and end-effectors of the master and slave robots under the action of the controllers presented in [12], respectively. In experiment case 1, an improved P + d control scheme in [12] is applied to realize the position tracking of the master and slave robots. It can be seen from Figure 13 that the gravity torque compensation is not applied in the position tracking control, which results in the tracking curves of the three active joints having large tracking errors with the action of external forces. The unsatisfactory end-tracking performance in Figure 14 also verifies the above analysis.

In experiment case 2, an adaptive control method in [23] is also employed to realize the position tracking of the master and slave robots. It can be seen from Figure 13 and Figure 15 that the adaptive method can compensate for the gravity torque and external forces; the joint tracking errors of the master and slave robots are also reduced. However, there are still the position tracking errors compared with Figure 11. It can be seen from the comparison between Figure 12 and Figure 16 that the method proposed in this paper has smaller tracking errors, and also has more performance advantages in terms of the position tracking of the robot end-effectors. These two comparative environments verify the effectiveness of the control scheme proposed in our paper.

## 6. Conclusions

This paper presents a new bilateral adaptive control scheme for the teleoperation system. The adaptive controller consists of an auxiliary variable, a nominal model controller, and an RBF neural network adaptive controller. The velocity feedback filter and integral of tracking error are applied to design the auxiliary variable. RBF neural network is used to estimate the uncertain parts of the system. The proposed control scheme can effectively achieve position tracking and eliminate the adverse effects of time-varying delays and dynamic uncertainties. Furthermore, compared with some related work, our method does not need to calculate the position acceleration signals. The simulation and experiment results are given to verify the effectiveness of the proposed control structure.

## Figures and Tables

**Table 1:** RMSE of position tracking errors with different control methods.

RMSE	Proposed	Method 1	Method 2	Method 3
$e_{m1}$	**0.005325**	0.018781	0.018986	0.066509
$e_{m2}$	**0.005216**	0.021510	0.033383	0.065783
$e_{s1}$	**0.002939**	0.015770	0.019065	0.041126
$e_{s2}$	**0.002333**	0.017474	0.028411	0.040475

## Data Availability

Not applicable.
